# Nitrate of Potass in Asthma

**Published:** 1844-03-09

**Authors:** 


### NITRATE OF POTASS IN ASTHMA.

A correspondent of the New York Med. Gaz., says that he has derived essential benefit from using the following remedy in severe attacks of asthma, and has prescribed it for several patients with equal success:—Immerse thick porous paper in a saturated solution of nitrate of potass, or common saltpetre, and hang it up to dry. At the approach of a paroxysm, inhale the vapour by burning it in the room, or smoking it in a tobacco-pipe.

